# Metabolic syndrome and the short-term prognosis of acute ischemic stroke: a hospital-based retrospective study

**DOI:** 10.1186/s12944-015-0080-8

**Published:** 2015-07-22

**Authors:** Liu Liu, Lixuan Zhan, Yisheng Wang, Chengping Bai, Jianjun Guo, Qingyuan Lin, Donghai Liang, En Xu

**Affiliations:** Institute of Neurosciences and the Second Affiliated Hospital of Guangzhou Medical University; Key Laboratory of Neurogenetics and Channelopathies of Guangdong Province and the Ministry of Education of China, 250 Changgang Dong RD, Guangzhou, 510260 People’s Republic of China; Department of Environmental Health Sciences, Rollins School of Public Health, Emory University, 1518 Clifton Road, 2040K, Atlanta, GA 30322 USA

**Keywords:** Metabolic syndrome, Acute ischemic stroke, Hyperglycemia, Prognosis

## Abstract

**Background:**

Metabolic syndrome (MetS) is an important risk factor for cerebral ischemic stroke, yet previous studies on the relationship between MetS or its components and acute cerebral infarction have been inconsistent. This study aims to evaluate the effects of MetS and its components on the short-term prognosis of patients with acute ischemic stroke.

**Methods:**

Subjects with ischemic stroke of <7-day duration (530 cases) were enrolled. MetS was defined based on the modified criteria of the International Diabetes Federation and the American Heart Association/National Heart, Lung, and Blood Institute. Demographic data, vascular risk factors, National Institutes of Health Stroke Scale score, the results of physical, laboratory and imaging examinations and clinical outcomes at 30 and 90 days were recorded. Using univariate analysis, we compared different baseline characteristics between patients with MetS and those without MetS. Further, we assessed MetS and its 5 components on the contribution to short-term prognosis of ischemic stroke with multiple logistic regression models after adjusting for age and sex.

**Results:**

The prevalence of MetS among the patients with acute ischemic stroke in the study is 58.3 %, with more in females (70.3 %) than in males (49.7 %, *p* < 0.001). As expected, among the MetS components, elevated waist circumference, elevated triglyceride, high fasting blood glucose and low high density lipoprotein cholesterol (HDL-C) were significantly more prevalent in patients with MetS than those without MetS (all *p* < 0.001). There was no correlation between MetS itself and the short-term prognosis of acute ischemic stroke. Only hyperglycemia in the serum was shown to have impact on poor functional outcomes in 30 and 90 days after the onset of stroke.

**Conclusions:**

The occurrence of MetS among patients with acute ischemic stroke in our study is 58.3 %. MetS itself may not be predictive for the short-term prognosis of patients, while hyperglycemia is a significant predictor for poor functional outcomes in our study.

## Background

Stroke has been the most important cause for disability and clinical death because of its high prevalence, morbidity and recurrence in China [1]. Multiple risk factors are involved in the progress of acute cerebrovascular diseases, including unmodifiable risk factors, such as age, gender, race/ethnicity and heredity, and modifiable risk factors, such as hypertension, diabetes mellitus, atrial fibrillation and other cardiac diseases, dyslipidemia, asymptomatic carotid stenosis, obesity, unhealthy lifestyle, metabolic syndrome (MetS) etc.

MetS can be defined as a constellation of interrelated metabolic abnormalities that increases the risk of cerebrovascular diseases. It includes insulin resistance/diabetes, elevated blood pressure, obesity, and dyslipidemia [2]. The insulin resistance is deemed as the primary pathomechanism of MetS, according to World Health Organization diagnostic criteria [3]. Numerous epidemiologic and clinical studies indicated that MetS was highly correlated to the occurrence of stroke [4–6], even recurrence [7, 8]. Persons with MetS have a significantly higher risk of incident ischemic stroke than those with no MetS. After adjusting other risk factors, the number of MetS components is highly correlated to the risk of ischemic stroke incidence [9, 10]. However, less information is available in China on the potential relationship between MetS including its components and short-term outcomes of acute ischemic stroke. Thus, the present study aims to investigate the effects of MetS and its individual components on ischemic stroke, and their impact on the short-term outcomes of acute ischemic stroke among our hospitalized patients.

## Results

### Baseline characteristics of the patients

Baseline characteristics of acute ischemic patients with or without MetS are presented in Table 1. Five hundred and thirty participants (222 women) with the average age of 69.3 ± 11.2 (from 30 to 94 years old) were enrolled in this study, in which 309 (58.3 %) patients met criteria for MetS, and the occurrence of MetS in females was 70.3 % (156/222), and that of males is 49.7 % (153/308) (*p* < 0.001). The proportions of smokers and drinkers were less frequent in patients with MetS than in patients without MetS (*p* = 0.001 and 0.002 respectively). As expected, patients with MetS had more prevalent family history of hypertension and diabetes mellitus (*p* < 0.001). In MetS group, history of medication use for hypertension and diabetes mellitus were significantly more prevalent than that in non-MetS group (*p* < 0.001). Moreover, among the five MetS components, elevated waist circumference (WC), high fasting blood glucose (FBG), high serum triglyceride (TG) and low high density lipoprotein cholesterol (HDL-C) were significantly more prevalent in the MetS group (*p* < 0.001). In addition, body mass index (BMI), serum total cholesterol (TC), lipoprotein cholesterol (LDL-C), uric acid (UA) and fibrinogen were significantly higher for patients with MetS, compared to the patients without MetS (all *p* < 0.05). There were no difference found between these two groups regarding blood pressure, neurologic impairment (National Institutes of Health Stroke Scale (NIHSS) scores), temperature on admission and delayed time prior to admission and common carotid arteries (CCAs) intima-media thickness (IMT).

**Table 1:** Characteristics of patients with MetS^+^ and MetS

Characteristic	MetS^+^	MetS^−^	*P* value
*n*	309	221	
Age (years)	68.8 ± 10.2	69.9 ± 12.4	0.248
Female, *n* (%)	156 (50.5)	66 (29.9)	<0.001
Smoker, *n* (%)	104 (33.7)	107 (48.4)	0.001
Drinker, *n* (%)	39 (12.6)	51 (23.1)	0.002
Pre-history, *n* (%)			
HTN	225 (72.8)	118 (53.4)	<0.001
DM	82 (26.5)	10 (4.5)	<0.001
CHD	25 (8.1)	21 (9.5)	0.275
TIA	8 (2.6)	9 (4.1)	0.339
Stroke	107 (34.6)	65 (29.4)	0.206
Treatment history, *n* (%)			
Anti-HTN	201 (65.0)	95 (43.0)	<0.001
Anti-DM	75 (24.3)	8 (3.6)	<0.001
Family history, *n* (%)			
HTN	108 (35.0)	53 (24.0)	0.007
DM	20 (6.5)	5 (2.3)	0.024
CHD	2 (0.6)	0 (0.0)	0.631
CVD	19 (6.1)	16 (7.2)	0.618
WC (cm)			
Male	91.9 ± 7.2	82.9 ± 7.2	<0.001
Female	89.9 ± 9.2	82.9 ± 10.5	<0.001
BMI (kg/m^2^)	24.2 ± 3.2	21.4 ± 3.3	<0.001
SBP (mmHg)	151.8 ± 23.3	148.5 ± 23.6	0.109
DBP (mmHg)	86.1 ± 12.1	84.8 ± 12.5	0.232
Median NIHSS	4 (2 ~ 9)	4 (2 ~ 9)	0.562
Temperature (°C)	36.6 ± 0.5	36.6 ± 0.5	0.240
Prehospital delay (d)	6 (4, 48)	19 (4, 48)	0.128
Laboratory data			
TG (mmol/L)	1.35 (1.02 ~ 1.90)	0.95 (0.74 ~ 1.17)	<0.001
TC (mmol/L)	5.41 ± 1.30	5.09 ± 1.20	0.004
HDL-C (mmol/L)	1.08 ± 0.29	1.35 ± 0.35	<0.001
Male	0.99 ± 0.28	1.28 ± 0.33	<0.001
Female	1.17 ± 0.27	1.54 ± 0.31	<0.001
LDL-C (mmol/L)	3.62 ± 1.08	3.29 ± 1.06	0.001
Apo A, mmol/L	1.07 ± 0.22	1.18 ± 0.23	0.380
ApoB, mmol/L	1.07 ± 0.28	0.94 ± 0.27	0.928
FBG (mmol/L)	5.74 (4.91 ~ 6.95)	4.73 (4.27 ~ 5.27)	<0.001
Creatinine (umol/L)	100.07 ± 35.53	98.69 ± 30.04	0.640
BUN (mmol/L)	5.34 ± 2.10	5.31 ± 2.22	0.854
UA (umol/L)	346.71 ± 134.53	324.56 ± 11.22	0.039
Fibrinogen (g/L)	3.63 ± 2.93	3.09 ± 1.20	0.011
CCA IMT (mm)	0.58 ± 0.50	0.56 ± 0.48	0.093

Data are acquired on admission and presented as means ± SDs or median (25th to 75th percentiles) for all quantitative traits. The unpaired Student’s *t*-test or Mann-Whitney test for continuous variables and the chi-square test or Fisher’s exact test for categorical variables were used to compare the values of MetS and non-MetS patients

*HTN* hypertention, *DM* diabetes mellitus, *CHD* coronary heart disease, *TIA* transient ischemic attack, *CVD* cerebral vascular disease, *WC* waist circumference, *BMI* body mass index, *SBP* systolic blood pressure, *DBP* diastolic blood pressure, *TG* triglyceride, *TC* total cholesterol, *HDL*-*C* high density lipoprotein cholesterol, *LDL-C* low density lipoprotein cholesterol, *ApoA* Apolipoprotein A, *ApoB* Apolipoprotein B, *FBG* fasting blood glucose, *BUN* blood urea nitrogen, *UA* uric acid, *CCA IMT* common carotid artery intima media thickness

### Association of MetS and individual components with short-term outcomes of the patients

At 30 days after the onset, 229 patients (modified Rankin Scale (mRS) ≥3) or 231 patients (Barthal Index (BI) <60) were identified with poor functional outcomes, and 23 patients died, and 31 patients suffered from recurrence. Also, at 90 days after the onset, 221 patients (mRS ≥3) or 226 patients (BI <60) were identified with poor functional outcomes, and 39 patients died, and 51 patients suffered from recurrence. The results from univariate binary logistic regression analysis are presented in Table 2. There were no significant associations between MetS and stroke short term outcomes. However hyperglycemia was found to be a predictor for poor functional outcome and death whenever at 30 or 90 days after the onset. No component was found to account for stroke recurrence. However, low HDL-C was identified as a decreased risk for poor functional outcome at 90 days after the onset. Unexpected, elevated TG was also found to be a decreased risk for poor functional outcome whenever at 30 or 90 days after the onset. In addition, there was no dose-response relationship between the numbers of MetS components and short-term prognosis of ischemic stroke in this study as well.

**Table 2:** Impact of MetS and its individual components on stroke short-term outcomes

		MetS			Hyperglycemia			Hypertension			Low HDL-C			Elevated TG			Elevated WC			No. of Components		
	*n*	*p*	OR	95 % CI	*p*	OR	95 % CI	*p*	OR	95 % CI	*p*	OR	95 % CI	*p*	OR	95 % CI	*p*	OR	95 % CI	*p*	OR	95 % CI
30 days after the onset																						
mRS ≥3	229	0.788	0.95	0.64 ~ 1.35	<0.001	2.03	1.02 ~ 1.06	0.847	0.85	0.47 ~ 1.53	0.12	0.76	0.54 ~ 1.07	0.001	0.46	0.29 ~ 0.72	0.485	0.87	0.59 ~ 1.28	0.568	0.96	0.82 ~ 1.11
BI <60	231	0.514	1.12	0.79 ~ 1.59	<0.001	2.10	1.48 ~ 2.99	0.618	0.86	0.48 ~ 1.55	0.12	0.76	0.53 ~ 1.08	<0.001	0.40	0.25 ~ 0.64	0.224	0.35	0.99 ~ 1.03	0.4.02	1.06	0.92 ~ 1.24
Death	23	0.805	1.16	0.44 ~ 2.85	0.010	3.34	1.33 ~ 8.35	0.014	0.25	0.09 ~ 0.76	0.612	1.25	0.52 ~ 2.99	0.064	0.15	0.02 ~ 1.12	0.647	0.80	0.31 ~ 2.05	0.608	0.91	0.63 ~ 1.31
Recurrence	31	0.084	0.52	0.24 ~ 1.09	0.801	0.91	0.43 ~ 1.94	0.957	0.97	0.28 ~ 3.36	0.35	1.43	0.68 ~ 3.01	0.156	0.41	0.12 ~ 1.41	0.157	0.57	0.27 ~ 1.24	0.221	0.82	0.59 ~ 1.13
90 days after the onset																						
mRS ≥3	221	0.387	0.86	0.60 ~ 1.22	<0.001	2.01	1.41 ~ 2.85	0.436	0.79	0.44 ~ 1.43	0.030	0.68	0.48 ~ 0.96	0.002	0.49	0.33 ~ 0.78	0.262	0.80	0.54 ~ 1.18	0.334	0.93	0.80 ~ 1.08
BI <95	226	0.502	1.13	0.80 ~ 1.60	<0.001	2.12	1.49 ~ 3.02	0.523	0.83	0.46 ~ 1.49	0.092	0.74	0.53 ~ 1.05	0.002	0.50	0.31 ~ 0.78	0.212	0.78	0.53 ~ 1.15	0.531	1.05	0.90 ~ 1.22
Death	39	0.225	0.63	0.30 ~ 1.33	0.025	2.18	1.10 ~ 4.31	0.099	0.45	0.17 ~ 1.16	0.855	1.07	0.54 ~ 2.11	0.061	0.31	0.09 ~ 1.05	0.097	0.55	0.28 ~ 1.11	0.223	0.83	0.62 ~ 1.11
Recurrence	51	0.984	1.02	0.56 ~ 1.86	0.775	0.92	0.51 ~ 1.66	0.870	0.92	0.35 ~ 2.46	0.248	1.42	0.78 ~ 2.59	0.698	1.15	0.57 ~ 2.35	0.708	0.88	0.45 ~ 1.71	0.611	1.07	0.83 ~ 1.38

Univariate binary logistic regression analysis was used to assess following variables, MetS, hyperglycemia, hypertension, low HDL-C, elevated TG and high WC to the contribution of stroke prognosis

*MetS* metabolic syndrome, *HDL-C* high density lipoprotein cholesterol, *TG* triglyceride, *WC* waist circumference, *mRS* modified Rankin Scale, *BI* barthal index, *OR* odds ratios, *CI* confidence interval

In multivariate logistic regression analysis after adjusting for age and sex, hyperglycemia was identified as an independent risk factor for poor functional outcome whenever at 30 or 90 days after the onset. The odds ratios (OR) of hyperglycemia were 2.08 (95 % confidence interval (CI): 1.46–2.96) for functional outcome when assessed with mRS ≥3 and 2.07 (95 % CI: 1.45–2.97) with BI <60 at 30 days after stroke onset respectively. For poor functional outcome at 90 days after the onset, the OR of hyperglycemia were 2.00 (95 % CI: 1.40–2.84) when assessed with mRS ≥3 and 2.12 (95 % CI: 1.47–3.04) with BI <60 respectively. Moreover, we reconfirmed that both low HDL-C and elevated TG decrease the risks for poor functional outcome whenever at 30 or 90 days after the onset with multivariate regressive analysis after adjusting for age and sex (Table 3).

**Table 3:** Impact of MetS and its individual components on stroke short-term outcomes, adjusted for age and sex

		MetS			Hyperglycemia			Hypertension			Low HDL-C			Elevated TG			Elevated WC			No. of Components		
	*n*	*p*	OR	95 % CI	*p*	OR	95 % CI	*p*	OR	95 % CI	*p*	OR	95 % CI	*p*	OR	95 % CI	*p*	OR	95 % CI	*p*	OR	95 % CI
30 days after the onset																						
mRS	229	0.708	0.93	0.65 ~ 1.35	<0.001	2.08	1.46 ~ 2.96	0.051	0.55	0.30 ~ 1.00	0.055	0.70	0.49 ~ 1.01	0.003	0.49	0.31 ~ 0.79	0.717	0.93	0.61 ~ 1.40	0.474	1.06	0.90 ~ 1.24
BI	231	0.388	0.85	0.59 ~ 1.23	<0.001	2.07	1.45 ~ 2.97	0.167	0.64	0.34 ~ 1.20	0.048	0.69	0.48 ~ 0.99	<0.001	0.43	0.27 ~ 0.69	0.333	0.82	0.54 ~ 1.23	0.281	1.09	0.93 ~ 1.28
Death	23	0.660	1.23	0.50 ~ 3.03	0.003	2.59	1.07 ~ 6.28	0.012	0.25	0.09 ~ 0.74	0.863	0.93	0.39 ~ 0.98	0.124	0.20	0.03 ~ 1.54	0.976	0.99	0.37 ~ 2.62	0.739	1.07	0.72 ~ 1.58
Recurrence	31	0.271	0.66	0.31 ~ 1.39	0.586	0.81	0.39 ~ 1.72	0.739	0.81	0.23 ~ 2.81	0.685	1.17	0.55 ~ 2.46	0.181	0.44	0.13 ~ 1.47	0.164	0.57	0.26 ~ 1.26	0.239	1.22	0.88 ~ 1.71
90 days after the onset																						
mRS	221	0.404	0.86	0.60 ~ 1.23	<0.001	2.00	1.40 ~ 2.84	0.030	0.51	0.28 ~ 0.94	0.015	0.63	0.44 ~ 0.91	0.011	0.55	0.34 ~ 0.87	0.605	0.90	0.59 ~ 1.36	0.345	1.08	0.92 ~ 1.27
BI	226	0.466	0.87	0.60 ~ 1.26	<0.001	2.12	1.47 ~ 3.04	0.100	0.59	0.31 ~ 1.11	0.042	0.68	0.47 ~ 0.99	0.010	0.55	0.34 ~ 0.87	0.420	0.84	0.56 ~ 1.27	0.472	1.06	0.90 ~ 1.25
Death	39	0.578	0.82	0.41 ~ 1.65	0.082	1.82	0.93 ~ 3.56	0.058	0.39	0.15 ~ 1.03	0.676	0.86	0.42 ~ 1.73	0.121	0.38	0.11 ~ 1.29	0.301	0.68	0.33 ~ 1.42	0.412	1.14	0.84 ~ 1.55
Recurrence	51	0.902	1.04	0.57 ~ 1.90	0.755	0.911	0.51 ~ 1.64	0.758	0.86	0.32 ~ 2.30	0.220	1.45	0.80 ~ 2.62	0.479	1.28	0.64 ~ 2.56	0.965	0.99	0.50 ~ 1.94	0.573	0.93	0.72 ~ 1.21

Multiple logistic regression models were conducted to assess following variables, MetS, hyperglycemia, hypertension, low HDL-C, elevated TG and high WC to the contribution of stroke prognosis after adjustment for age and sex

*MetS* metabolic syndrome, *HDL-C* high density lipoprotein cholesterol, *TG* triglyceride, *WC* waist circumference, *mRS* modified Rankin Scale, *BI* barthal index, *OR* odds ratios, *CI* confidence interval

## Discussion

The prevalence of MetS in China has been steadily rising in recent decades and reached 58.1 % in elderly Chinese population [11]. A longitudinal study from Beijing Tongren Hospital showed that the 5-year cumulative incidence of MetS was 10.82 % in 2007 to 2012 [12]. In the United States, the prevalence of MetS was 38.5 % in adults [13] and it is 21.1 % in the French population [14]. As a potential risk factor, MetS has been associated with an increased risk of prevalent stroke [6]. The data from a meta-analysis about 13 cohort studies showed that subjects with MetS were 1.6-fold more likely to have stroke than the ones without MetS [15] The occurrence of MetS among the patients with acute ischemic stroke in our study is 58.3 %. In the other two acute ischemic stroke studies of Chinese, the occurrence of MetS was 51.4 and 57.29 %, respectively [8, 16]. There were more female (70.3 %) than male (49.7 %) stroke patients with MetS, which is consistent with the results from the other study [4], and there were less stroke patients with smoking or drinking in MetS^+^ group. However, the previous studies tend to exhibit causal correlation between MetS and smoking and heavy alcohol consumption [17, 18]. We are unsure about why the result of our current study differs from that of previous ones; it may be due to the small sample size of patients enrolled. Other risk factors were further compared between the two major stroke groups with or without MetS. In this study, we found that there were positive and significant differences in the components of MetS (high WC, elevated TG and depressed HDL-C, hypertension and hyperglycemia) between two groups.

Dyslipidemia (elevated TG, decreased HDL-C and increased LDL-C) and central obesity would enhance the development and progress of cerebrovascular atherosclerosis, especially occlusive large artery diseases [19]. Hypertension is the most important risk factor for all stroke subtypes [20, 21]. It is known that insulin resistance is a crucial factor to cause multiple proatherombotic effects on the fibrinolytic system and vascular endothelium [22].

Numerous studies on the MetS and ischemic stroke have been evaluated in the field of stroke prevention, but the data about the impact of MetS and its individual components on acute stroke prognosis is limited. Our results agree with recent studies that MetS components such as hyperglycemia are associated with the poor short-term outcomes [7, 16, 23, 24].

Our results also showed that hyperglycemia is an independent predictor for patient’s severe disability at 30 and 90 days rather than MetS itself, based on the multiple logistic regression analysis after adjusting for age and sex. Hyperglycemia was positively associated with the functional outcome of acute ischemic stroke [25–28]. Several explanations may account for the association between hyperglycemia and poor functional outcomes after ischemic stroke. First, hyperglycemia may be directly toxic to the ischemic neurons. In an experimental stroke model, hyperglycemia aggravated the cellular acidosis in the ischemic penumbra and resulted in a greater infarct volume [29]. Second, hyperglycemia may disrupt the blood-brain barrier [30] and promote hemorrhagic infarct conversion [31]. Third, hyperglycemia may be as a marker of severe ischemic damage in patients with stroke. Patients with severe stroke might develop hyperglycemia due to greater release of stress hormones such as cortisol. These results highlight the need for further study to determine whether glucose lowering after stroke can improve functional outcomes of ischemic stroke patients.

As for the additional implicative components for ischemic stroke, serum low HDL-C and high TG have negative effects on 30 and/or 90 days prognosis in this study. However, cumulative data supported the idea that dyslipidemia (low HDL-C and high TG) was directly related to the poor outcomes of ischemic stroke patients [7, 32]. The main reason for our paradoxical results might be due to the fact that we did not control for all the other confounding factors. Also, HDL-C particles can be assumed to have pro-inflammatory and pro-atherogenic properties especially in the condition of chronic systemic inflammatory response [33]. In addition, HDL-C increases formation of lipid peroxide and oxidation of LDL-C and phospholipids in patients with a history of coronary heart diseases [34]. Further studies with large sample size are needed to investigate these associations comprehensively.

Similar with other studies [8], there was no evidence in present study to show that MetS was associated with recurrence and poor outcomes of acute ischemic stroke. Theoretically, MetS might have aggravated or deteriorated patients’ condition and prognosis. MetS-related impairments comprise hyperglycemia, chronic endothelial damage, decreased endogenous fibrinolytic capacity, and proinflammatory state, all of which may amplify cerebral ischemic damage and to hamper arterial recanalization [35]. However, there was no similar phenomenon in our study. This might be explained by the following two reasons: a) limited sample volume; and b) this study presented an offset effect of MetS components which showed a positive effect in hyperglycemia but negative in hypertension, low HDL-C and elevated TG on the short term outcomes of cerebral infarction. These components might offset each other, which may even lead to irrelevance between MetS and stroke short-term prognosis. Additionally, no dose-response relationship between the numbers of MetS components and short-term prognosis of ischemic stroke was found in this study.

There were several limitations in our study. First, our data was based on one hospital-based cohort, and the small sample size may not be appropriate to generalize. Second, although the patients with acute ischemic stroke admitted to the Department of Neurology were consecutively registered, the patients who were sent to the Emergency Department and died soon there before hospitalization were excluded. Therefore, it is possible that the case fatality and other poor outcomes from our study were under-estimated to some extent. Third, no data was gathered on long-term outcomes for these patients. Despite these limitations, our pilot study did provide some important information about the occurrence of MetS and the effects of MetS and its components on the short-term prognosis of patients with acute ischemic stroke. We believe that it would be useful for further study because MetS and its components are modifiable risk factors for ischemic stroke. Above all, a multi-center prospective cohort study is needed to further investigate the relationship between MetS and stroke for its primary and secondary prevention.

## Conclusion

The occurrence of MetS among patients with acute ischemic stroke in our study is 58.3 %. MetS itself may not be predictive of the short-term prognosis of patients, while hyperglycemia is a significant predictor for poor functional outcomes. Our data provided valuable information toward better understanding of individuals who are at increased risk of ischemic stroke and reaffirmed the need to develop preventive strategies directed to the control of MetS and each of its component conditions for future stroke.

## Methods

### Subjects

**Fig. 1:**
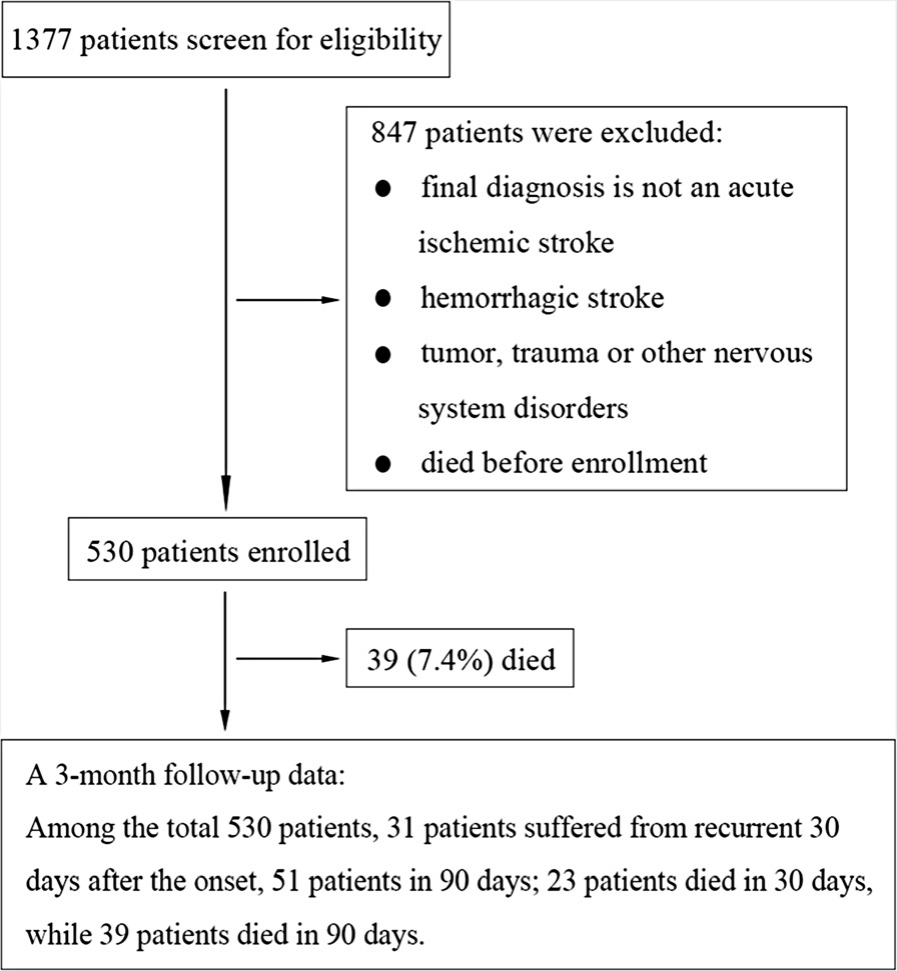
Study design

A hospital-based retrospective study was performed from January 1, 2006 to December 31, 2006. During this period, a total of 1377 consecutive stroke patients were admitted to the Department of Neurology, the Second Affiliated Hospital of Guangzhou Medical University. Five hundred and thirty patients with acute ischemic stroke (within 7 days from symptom onset) were eligible to be recruited in this study (Fig. 1). Criteria for exclusions were patients who experienced the onset of stroke more than 7 days before hospitalization, or who were diagnosed with “silent stroke” or who were sent to the Emergency Department and died soon there before hospitalization. Patients with cerebral hemorrhage, subarachnoid hemorrhage, brain tumor or other central nervous system disorders were also excluded. This study was approved by the Hospital Institutional Ethics Committee. Also, written informed consent to participate in the study was obtained from each patient or his/her relative if patients could not consent by themselves.

All the subjects presented clinical characteristics of acute ischemic stroke, confirmed by cranial computed tomography and/or magnetic resonance imaging/angiography.

### Assessments

Data collection was performed by using a standardized questionnaire based on an extensive manual and follow-up information. General information, present illness, previous history (including hypertension, diabetes mellitus, coronary heart disease, transient ischemic attack and stroke), personal history (including cigarette smoking and drinking habits), family history (including hypertension, diabetes mellitus, coronary heart diseases and cerebrovascular diseases), the data of physical examination, laboratory and imaging results were all recorded for all subjects enrolled in this study. Carotid ultrasonography, transcranial doppler and magnetic resonance angiography were used to evaluate brain-supplying arteries. Cardiac diagnostic test such as electrocardiography and transthoracic echocardiography were used to identify cardioembolic stroke.

All the baseline clinical characteristics were recorded at the time of admission while venous blood samples were extracted after 12 h fasting time at the second day of hospitalization. Serum glucose, TG, TC, HDL-C, LDL-C, apolipoprotein (Apo)-A, Apo-B, creatinine, urea nitrogen and UA were measured by Hitachi 7600 automatic analyzer (Hitachi Instruments Corporation, Tokyo, Japan). Blood fibrinogen was evaluated by turbidimetry with the use of CA7000 (Sysmex, Japan).

Waist circumference was measured by a measuring tape positioned at the narrowest part between the lowest rib margin and the high point of the iliac crest after a normal expiratory breath. Body height was measured, without wearing shoes, with an accuracy of 0.1 cm, using a calibrated stadiometer. Body weight was measured to the nearest 0.1 kg, wearing underwear, with a calibrated electronic scale. BMI was calculated by dividing weight by height squared (kg/m^2^). Blood pressure was measured on the right arm at heart level with a mercury sphygmomanometer after being seated for at least 5 min. CCAs IMT was defined as the distance between the edges of the lumen-intima interface and the media-adventitia interface of the far wall. In this study, IMT of both CCAs were uniformly measured by one sonographer, using color doppler ultrasonography (ALOKA prosound α5, Hitachi Instruments Corporation, Tokyo, Japan) with a 7.5 ~ 10.0 MHz transducer frequency in 3 days after admission.

Clinical assessments consisted of the NIHSS, mRS and BI, which were performed by four well-trained neurologists who were blinded to magnetic resonance imaging results. The score of NIHSS is from 0 (normal) to 42, measured at the admission. Functional outcome was assessed with mRS and BI at 30 and 90 days after the occurrence of stroke. Patients who died scored 6 in the mRS. A mRS of 0 to 2, or a BI score of 95 to 100 was considered as a favorable outcome, and mRS ≥3 or BI <60 was used as cutoff scores to defined poor outcome [36].

Recurrent stroke was defined as any new episode of focal cerebral dysfunction persisting >24 h, which occurred after a period of unequivocal neurological stability or improvement. This definition excluded any new deficit that occurred within 24 h or that was thought to be attributable to edema, mass effect, brain shift syndrome, or hemorrhagic transformation of the incident infarct.

Patients were followed up with hospital visits during the first and three months after the stroke event. Patients who were unable to attend the scheduled visits or had migrated from our city were contacted by telephone. In case of death, dates and causes were registered by gathering information from relatives or records kept by hospitals.

### Definition of metabolic syndrome

Patients were classified into two groups at baseline based on whether or not the diagnostic criteria for MetS were met. According to the American Heart Association/National Heart, Lung, and Blood Institute definition for MetS [37], MetS was defined as the presence of any three of the following five risk components: (i) elevated WC (male ≥ 85 cm, female ≥ 80 cm), (ii) TG ≥ 1.70 mmol/L, (iii) HDL-C < 1.0 mmol/L (male) or <1.3 mmol/L (female), (iv) hypertension: systolic pressure ≥ 130 mmHg, diastolic pressure ≥ 85 mmHg or need for anti-hypertensive medication and (v) hyperglycemia: FBG ≥ 5.6 mmol/L (≥100 mg/dL) or need for anti-hyperglycemic medication.

### Statistical analysis

The data was entered duplicatedly into a stroke data bank built with Microsoft Visual FoxPro 6.0 and checked for completeness, logical consistency and duplication, and then locked in. Statistical analyses were performed with the Statistical Package for Social Sciences for Windows, version 13.0 (SPSS, Inc, Chicago, IL, USA). Measurement data were summarized by mean ± standard deviation or as median and 25th and 75th quartile values. The differences of baseline characteristics of the patients were compared with the unpaired Student’s *t*-test or Mann-Whitney test for continuous variables and the chi-square test or Fisher’s exact test for categorical variables. To generate the odds ratio, univariate binary logistic regression analysis was conducted to assess MetS, hyperglycemia, hypertension, low HDL-C, elevated TG and high WC to the contribution of stroke prognosis. Further, multiple logistic regression models were conducted after adjusting for age and sex.

## Abbreviations

Apo: Apolipoprotein
BI: Barthal index
BMI: Body mass index
CCAs: Common carotid arteries
CI: Confidence interval
IMT: Intima-media thickness
FBG: Fasting blood glucose
HDL-C: High density lipoprotein cholesterol
LDL-C: Lipoprotein cholesterol
MetS: Metabolic syndrome
mRS: modified Rankin Scale
NIHSS: National Institutes of Health Stroke Scale
OR: Odds ratios
TC: Total cholesterol
TG: Triglyceride
UA: Uric acid
WC: Waist circumference
